# Distribution, Evolution, Catalytic Mechanism, and Physiological Functions of the Flavin-Based Electron-Bifurcating NADH-Dependent Reduced Ferredoxin: NADP^+^ Oxidoreductase

**DOI:** 10.3389/fmicb.2019.00373

**Published:** 2019-03-01

**Authors:** Jiyu Liang, Haiyan Huang, Shuning Wang

**Affiliations:** ^1^State Key Laboratory of Microbial Technology, Microbial Technology Institute, Shandong University, Qingdao, China; ^2^Institute of Basic Medicine, Shandong Academy of Medical Sciences, Jinan, China

**Keywords:** flavin-based electron bifurcation, Nfn, gene fusion/fission, lateral gene transfer, structure, physiological function

## Abstract

NADH-dependent reduced ferredoxin:NADP^+^ oxidoreductase (Nfn) is an electron-bifurcating enzyme first discovered in the strict anaerobes *Clostridium kluyveri* and *Moorella thermoacetica*. *In vivo*, Nfn catalyzes the endergonic reduction of NADP^+^ with NADH coupled to the exergonic reduction of NADP^+^ with reduced ferredoxin. Most Nfn homologs consist of two subunits, although in certain species Nfn homologs are fused. In contrast to other electron-bifurcating enzymes, Nfn possess a simpler structure. Therefore, Nfn becomes a perfect model to determine the mechanism of flavin-based electron bifurcation, which is a novel energy coupling mode distributed among anaerobic bacteria and archaea. The crystal structures of Nfn from *Thermotoga maritima* and *Pyrococcus furiosus* are known, and studies have shown that the FAD molecule of the NfnB (b-FAD) is the site of electron bifurcation, and other cofactors, including a [2Fe2S] cluster, two [4Fe4S] clusters, and the FAD molecule on the NfnA subunit, contribute to electron transfer. Further, the short-lived anionic flavin semiquinone (ASQ) state of b-FAD is essential for electron bifurcation. Nfn homologs are widely distributed among microbes, including bacteria, archaea, and probably eukaryotes, most of which are anaerobes despite that certain species are facultative microbes and even aerobes. Moreover, potential evidence shows that lateral gene transfer may occur in the evolution of this enzyme. Nfn homologs present four different structural patterns, including the well-characterized NfnAB and three different kinds of fused Nfn homologs whose detailed properties have not been characterized. These findings indicate that gene fusion/fission and gene rearrangement may contribute to the evolution of this enzyme. Under physiological conditions, Nfn catalyzes the reduction of NADP^+^ with NADH and reduced ferredoxin, which is then used in certain NADPH-dependent reactions. Deletion of *nfn* in several microbes causes low growth and redox unbalance and may influence the distribution of fermentation products. It’s also noteworthy that different Nfn homologs perform different functions according to its circumstance. Physiological functions of Nfn indicate that it can be a potential tool in the metabolic engineering of industrial microorganisms, which can regulate the redox potential *in vivo*.

## Introduction

Flavin-based electron bifurcation (FBEB) is a novel mechanism of energy coupling found in anaerobes, which allows energy conservation via the membrane-bound ferredoxin:NAD^+^ reductase (Rnf) or [NiFe]-hydrogenase (Ech) complex (Buckel and Thauer, 2013, 2018b). It splits the hydride electron pairs into one electron with higher redox potential and the other with lower redox potential. Electron bifurcation amplifies the reducing power of one electron at the cost of that of the other electron (Buckel and Thauer, 2013, 2018a,b; Metcalf, 2016; Peters et al., 2016). The butyryl-CoA dehydrogenase/electron-transferring flavoprotein complex (Bcd/EtfAB) is the first enzyme known to contain a flavin that functions similarly to the quinone in the quinone-mediated electron bifurcation in the Q Cycle (Herrmann et al., 2008; Li et al., 2008; Chowdhury et al., 2014; Demmer et al., 2017). Eleven other flavoprotein complexes that employ this mechanism have been discovered in anaerobes subsequent to the discovery of the electron-bifurcating Bcd/EtfAB complex in *Clostridium kluyveri* (Buckel and Thauer, 2018b). These enzymes are involved in metabolic pathways such as butyric acid formation, CO_2_ fixation, H_2_ production, acetogenesis, and methanogenesis. Among them, acetogenesis and methanogenesis are regarded as two ancient biological processes. FBEB was therefore considered a mechanism through which ancient microbes conserved energy in an energy-limited environment (Nitschke and Russell, 2011; Martin et al., 2014). All known electron-bifurcating enzymes contain at least one flavin cofactor, FAD or FMN, which is regarded as the central component of electron bifurcation, therefore, the novel mechanism was defined to be flavin-based.

NADH-dependent reduced ferredoxin:NADP^+^ oxidoreductase (Nfn), also named ferredoxin-dependent transhydrogenase, is the third electron-bifurcating enzyme characterized in anaerobes. Its finding solved the old enigma that cell extracts of *C. kluyveri* produce H_2_ from NADPH under the regulation of NAD^+^/NADH (Jungermann et al., 1971). This complicated NADPH:ferredoxin oxidoreductase system was not identified until 40 years later (Wang et al., 2010). The special oxidoreductase comprises two subunits, named NfnAB, and catalyzes the reaction as follows:

Fdred2-+2NADP++NADH+H+ = Fdox+2NADPH +NAD+;ΔG0'                         = -20kJ/mol

In the complex, the 32.6 kDa NfnA binds one FAD molecule and one [2Fe2S] cluster, which shares sequence similarity with plant ferredoxin:NADP^+^ oxidoreductase. NfnB (49.8 kDa) shares amino acid sequence similarities with NADP^+^-dependent glutamate synthase and binds one FAD molecule and two [4Fe4S] clusters.

Soon after the discovery of NfnAB produced by mesophile *C. kluyveri*, its homologs in other anaerobes were characterized, such as the thermophilic acetogen *Moorella thermoacetica* (Huang et al., 2012), the hyperthermophile *Thermotoga maritima* (Demmer et al., 2015), and *Pyrococcus furiosus* (Lubner et al., 2017; Nguyen et al., 2017), all of which share similar properties.

Nfn possesses the simplest structure among known electron-bifurcating enzymes (Buckel and Thauer, 2018b). Therefore, Nfn is a perfect molecule to use as a model to elucidate the mechanism and function of electron bifurcation. Here, we review recent studies on the distribution, evolution, structure, catalytic mechanism, and physiological functions of Nfn.

## An Evolutionary View on Nfn

### Distribution of Nfn Homologs

**FIGURE 1:**
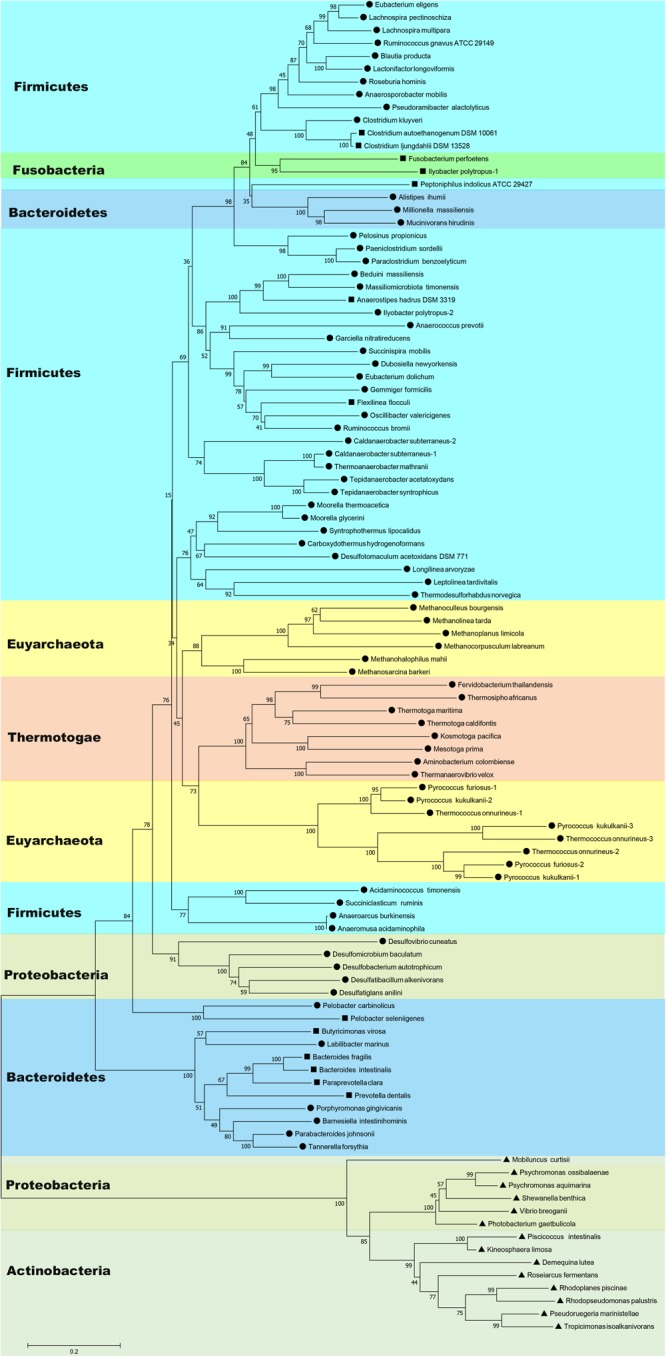
Neighbor-joining phylogenetic tree of Nfn homologs constructed using amino acid sequences. The connected sequences of NfnAB (Pattern A) and the full sequences of fused Nfn homologs (Pattern B and Pattern C) were used. Each branch is colored according to phylum and labeled according to their sequence pattern (circle for Pattern A, square for Pattern B, and triangle for Pattern C). Bootstrap values (out of 100) are shown at each branch point. Different copies of Nfn homologs in the same strain are presented with the number after the species name. Accession numbers of protein sequences of Nfn homologs are listed in the Supplementary Table S1.

BLAST searches have shown that Nfn homologs are widely distributed in bacteria and archaea, such as members of Firmicutes, Fusobacteria, Bacteroidetes, Thermotogae, Proteobacteria, Actinobacteria, Spirochaetes, and Euryarchaeota (Figure 1 and Supplementary Table S1). These homologs share sequences identities between 40 and 90% with the *C. kluyveri* NfnAB. Among 4,588 complete genomes of archaea, bacteria, and eukaryotes, 397 encode Nfn homologs, much more than those of other electron-bifurcating enzymes (Poudel et al., 2018). Their taxonomic distribution suggests that Nfn homologs are distributed in different environments and contribute to diverse metabolic pathways. In Firmicutes, the Nfn of *C. kluyveri* and *M. thermoacetica* have been characterized (Wang et al., 2010; Huang et al., 2012). Besides, many other *Clostridia* like *C. botulinum, C. sporogenes, C. autoethanogenum, C. ljungdahlii, C. saccharobutylicum*, and *Clostridioides difficile* [formerly named *Clostridium difficile* (Lawson et al., 2016)] also contain Nfn homologs, with one exception *C. acetobutylicum*. Some of them are acetogens (e.g., *M. thermoacetica* and *C. autoethanogenum*), which belong to arguably one of the most ancient groups of bacteria (Martin et al., 2014).

Further, Nfn is also encoded by species belonging to Bacteroidetes, Thermotogae, and Proteobacteria (Leavitt et al., 2016; Poudel et al., 2018). Among them, Bacteroidetes are a dominant groups in the human gut, along with Firmicutes, and may be associated with obesity (Ley et al., 2006). *T. maritima* was discovered in a geothermally heated marine sediment and metabolizes many simple and complex carbohydrates (Nelson et al., 1999). Nfn homologs are also present in most sulfate-reducing organisms, particularly Deltaproteobacteria species, which are ubiquitous in anoxic habitats. Among the sulfate-reducing organisms with sequenced genomes, all sulfate-reducing archaea and three sulfate-reducing bacteria (*Desulfovibrio aespoeensis, Desulfotalea psychrophila*, and *Thermodesulfovibrio yellowstonii*) lack apparent Nfn homologs (Pereira et al., 2011; Leavitt et al., 2016).

In archaea, the distribution of Nfn homologs appears to be restricted to the methanogenic order Methanomicrobia and the thermophilic Thermococci and Thermoprotei (Buckel and Thauer, 2013; Mccarver and Lessner, 2014; Poudel et al., 2018). Some archaea contain multicopy of *nfn*AB, which are all in the order Thermococcales. There are three isoforms of Nfn homologs in Thermococcales, namely, NfnI, NfnII, and NfnIII, and the NfnII-encoding genes are present in all the Thermococcales species with sequenced genomes (Nguyen et al., 2017).

Metagenomes data show that Nfn homologs are distributed among diverse environments (Poudel et al., 2018). Analysis of 3,136 metagenomes reveals that Nfn homologs are present in surface and subsurface environments and are enriched in subsurface environments, particularly in deep subsurface and hydrothermal vents. Among the enzymes encoded by metagenomes from different environments, Nfn is the most abundant electron-bifurcating enzyme (Poudel et al., 2018), suggesting that Nfn may be the most ubiquitous enzyme that employs FBEB. A metatranscriptomic study of methanogenic enrichment cultures derived from anaerobic cellulose or xylan digesters, shows that Nfn homologs are moderately transcribed among many genomes, including those of species of Clostridiaceae, Negativicutes, *Thermoanaerobacterium*, and Proteobacteria (Jia et al., 2018).

Most species containing Nfn homologs are strict anaerobes, although genomes of certain facultative microbes like *Enterococcus faecalis, Draconibacterium orientale*, and *Rhodopseudomonas palustris* encode Nfn homologs. Surprisingly, an Nfn homolog is present in the aerobe *Candidatus Koribacter versatilis* Ellin345 (protein locus tag: ABF41796.1) (Sait et al., 2002). This species is the only one with a putative electron-bifurcating enzyme homolog among the eight sequenced genomes of Acidobacteria species and the only aerobe known to harbor Nfn homologs (Poudel et al., 2018).

The wide distribution of Nfn in bacteria and archaea and their high abundance in subsurface environments indicate that Nfn homologs may play an important role in the anaerobic environment. Considering that acetogens and methanogens are thought as two of the most ancient microbes (Nitschke and Russell, 2011; Martin et al., 2014), the electron-bifurcating transhydrogenase Nfn may represent a crucial enzyme in the ancient energy metabolism. However, a recent phylogenetic reconstruction of Nfn suggested that Nfn may have a bacterial origin (Poudel et al., 2018). The rooted phylogenetic tree shows that the earliest evolving Nfn lineage comprises a homolog from phylum Actinobacteria (*Mobiluncus curtisii*, ADI67453) and the next closest branch comprises a homolog from Alphaproteobacteria (Poudel et al., 2018), suggesting that Nfn diversified from an ancestor of Actinobacteria and Alphaproteobacteria. Thus, the emergence of Nfn was supposed to follow the divergence of bacteria and archaea from Last Universal Common Ancestor (LUCA), and the wide distribution of Nfn homologs was proposed to be a result of lateral gene transfer (Poudel et al., 2018). These Nfn homologs from Actinobacteria and Alphaproteobacteria are encoded by one single gene and are significantly different from other Nfn homologs (see next section).

### Fusion/Fission and Duplication of *nfnAB*

**FIGURE 2:**
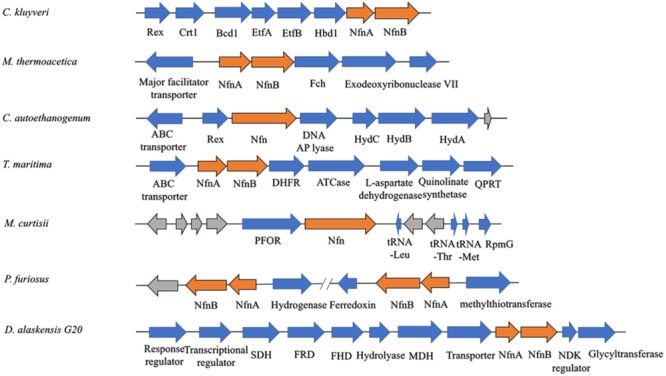
Gene arrangement of *nfn* homologs in several microbes. The *nfn* genes are in orange, other genes with putative products are blue, while genes with unknown product are drawn in gray. The *nfn* genes of different species are arranged in the same order and in different gene clusters that likely encode enzymes involved in different metabolic pathways. Rex, redox-sensing transcriptional repressor; Crt1, 3-hydroxybutyryl-CoA dehydratase; Bcd1, butyryl-CoA dehydrogenase; Etf, electron-transfer flavoprotein; Hbd1, NADP-dependent β-hydroxybutyryl-CoA dehydrogenase; Fch, methenyl-tetrahydrofolate cyclohydrolase; Hyd, bifurcating [FeFe]-hydrogenase; DHFR, dihydrofolate reductase; ATCase, aspartate carbamoyltransferase; QPRT, nicotinate-nucleotide pyrophosphorylase; PFOR, pyruvate:ferredoxin oxidoreductase; RpmG, ribosomal protein L33; SDH, succinate dehydrogenase; FRD, fumarate reductase; FHD, fumarate hydratase; MDH, malate dehydrogenase; NDK, nucleoside diphosphate kinase.

**FIGURE 3:**
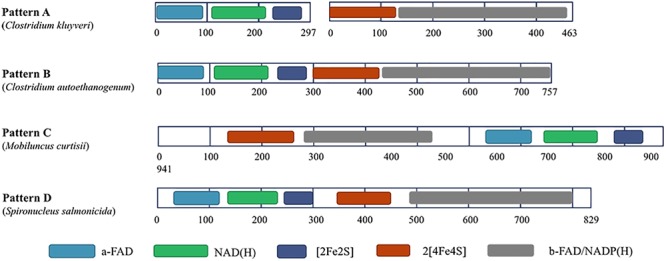
Different patterns of Nfn homologs. Conserved domains were identified using InterPro (https://www.ebi.ac.uk/interpro/) and shown in different colors. **Pattern A** (represented by NfnAB of *C. kluyveri*) is distributed throughout most species of bacteria and archaea. **Pattern B** (represented by Nfn homolog of *C. autoethanogenum*) is typical of the fused Nfn homologs of Firmicutes, Bacteroidetes, and Fusobacteria. **Pattern C** (represented by Nfn homolog of *M. curtisii*) characterizes the fused Nfn homologs of Actinobacteria, Alphaproteobacteria, and Gammaproteobacteria. **Pattern D** (represented by Nfn homolog of *Spironucleus salmonicida*) is distributed among few eukaryotic Nfn homologs of diplomonads.

Among most species encoding Nfn homologs, *nfnA* and *nfnB* are arranged in a conserved order (Figure 2), that is, *nfnA* is located on the upstream of the *nfnB* with a gap of a few base pairs, and in some certain cases, these genes overlap. Mantel test using pairwise distance of NfnA and NfnB shows a strong positive correlation, indicating these two subunits co-evolved (Poudel et al., 2018). In most anaerobes, NfnA and NfnB are encoded by two open reading frames; however, their homolog in *C. autoethanogenum* is encoded by a single gene (locus tag in GenBank: CAETHG_RS07665, 2,274 bp), whose sequence and length are very similar to those of *nfnAB* from *C. kluyveri* (Wang et al., 2010). This suggests that gene fusion or fission occurred during the evolution of *nfn*. BLAST analysis reveals that naturally fused Nfn is not specific to *C. autoethanogenum*, because similar fused genes are also present in genomes of other *Clostridium* species such as *C. ljungdahlii* and *C. ragsdalei*, which are close to *C. autoethanogenum* in both genome and metabolism. In members of the families Bacteroidaceae, Prevotellaceae, and Fusobacteriaceae, many Nfn homologs are encoded by similarly fused genes. Interestingly, Nfn homologs from certain species of Actinobacteria, Alphaproteobacteria, and Gammaproteobacteria are also naturally fused and are quite different from others. Moreover, compared with the fused Nfn homologs of *Clostridium* and Bacteroidaceae, such Nfn homologs are around 200 amino acid residues longer, and their cofactor binding domains do not structurally overlap. The binding domains of a-FAD, NAD, and the [2Fe2S] cluster are located in the C-terminal rather than N-terminal of these proteins. Thus, the known Nfn homologs could be grouped into four patterns (Figure 3). Pattern A is the classic pattern involved in NfnAB homologs, which has been well characterized. Pattern B is exhibited by the fused Nfn homologs from Firmicutes, Bacteroidetes, and Fusobacteria. Pattern C proteins represent the fused Nfn homologs from Actinobacteria and Proteobacteria, although there is another special Pattern D exhibited by eukaryotic Nfn homologs from diplomonads (see next section). These different patterns of Nfn homologs indicate that numerous gene fusions or fissions, and likely gene rearrangements may have contributed to the evolution of Nfn.

The genomes of certain species harbor more than one set of NfnAB homologs, which is more universal in thermophilic archaea like *Pyrococcus* and *Thermococcus*, in which most strains encode two or three sets of NfnAB homologs. According to their protein sequences, these Nfn homologs are classified into three branches called NfnI, NfnII, and NfnIII, respectively (Nguyen et al., 2017). The genomes of certain thermophilic bacteria such as *Thermoanaerobacter tengcongensis* and *T. italicus* also encode two sets of NfnAB homologs, however other thermophiles such as *Thermotoga* only have one set of NfnAB homolog. Moreover, certain mesophilic clostridia (e.g., *C. saccharolyticum, C. botulitum*, and *C. sporogenes*) and sulfur-reducing bacteria (e.g., *D. fructosovorans* and *D. alaskensis*) employ two or three sets of NfnAB homologs. There is no gene duplication of the naturally fused *nfn* in sequenced genomes.

### Potential Evidences for Lateral Gene Transfer

Evidence suggests the occurrence of lateral transfers of *nfn* between different groups of microbes. For example, the NfnAB homologs from *Desulfobulbus propionicus* and *Syntrophobacter fumaroxidans* are more similar to those of methanogenic archaea in contrast to their close family members, the sulfate-reducing bacteria of Deltaproteobacteria (Leavitt et al., 2016). These findings suggest that lateral gene transfer occurred between these two sulfate reducers and methanogens. The family Rikenellaceae represents a group of Bacteroidia whose Nfn sequences are more closely related to *Clostridium* species instead of other groups of Bacteroidia (Figure 1). Lateral gene transfer events from *Clostridium* species to the common ancestor of Rikenellaceae may explain this phenomenon. Further, in *Ilyobacter polytropus*, a member of phylum Fusobacteria, there is an extra copy of *nfnAB* on its plasmid (pILYOP01) along with the fused chromosomal *nfn*. The NfnAB homolog encoded by this plasmid is a relatively distant in evolution from its fused homologs from Fusobacteria and is more similar to the Nfn homologs of Firmicutes, thus, the plasmid-encoded *nfnAB* may have been acquired from another species. Although no other Nfn homologs are known to be encoded by plasmids, lateral transfer of *nfn* might have been achieved by a plasmid.

Interestingly, in the genome of a diplomonad, *Spironucleus salmonicida*, a sulfide dehydrogenase (Sud) is encoded (Xu et al., 2014), which shares little identity with eukaryotic proteins but shares 48% identity with the Nfn homolog from *C. autoethanogenum* (Pattern B). The eukaryotic Sud has a similar sequence pattern with the Pattern B (Figure 3), except that it is around 100 amino acids longer than the typical Pattern B Nfn homologs. Thus, we term the eukaryotic Sud from *S. salmonicida* as the Pattern D of Nfn homologs. Whether the activity of the eukaryotic Sud is similar to that of Nfn is unknown. In another diplomonad, *Giardia lamblia*, the Sud sequence was also found (Andersson and Roger, 2002), which shares 50 and 47% sequence identity with the Nfn of *M. thermoacetica* (Pattern A) and *S. salmonicida* (Pattern D). However, this Sud protein lacks the [2Fe2S] cluster binding domain, which is not likely to catalyze the Nfn reactions. The *sud* gene of diplomonads most likely arose from a prokaryote via lateral gene transfer. Further, compelling evidence indicates that the prokaryotic progenitor should be *nfn* (Andersson and Roger, 2002). NfnI of *P. furiosus* was once named as Sud since it exhibits sulfide dehydrogenase activity (Ma and Adams, 1994, 2001; Lubner et al., 2017), suggesting that the Sud of diplomonads may represent eukaryotic homologs of Nfn and share similar properties with bacterial and archaeal Nfn homologs.

## Structure and Catalytic Mechanism of Nfn

**FIGURE 4:**
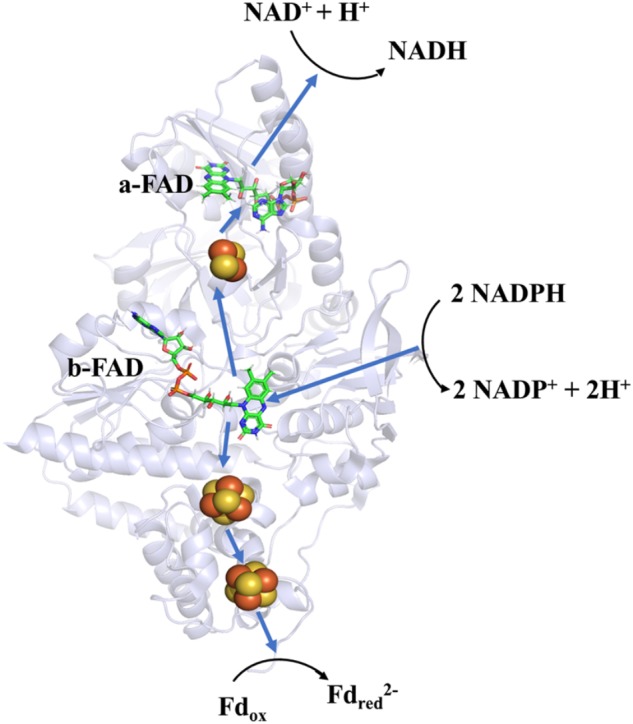
Overall structure and electron transfer pathway of *P. furiosus* NfnI (PDB: 5JCA). The overall structure of NfnI (light blue); iron–sulfur clusters are depicted by spheres (yellow, sulfur; orange, iron); FAD (green, carbon; blue, nitrogen; orange, oxygen; yellow, phosphorus). The blue arrows show the direction of electron transfer, and the black arrows indicates the reactions (Fd_red_, reductive ferredoxin; Fd_ox_, oxidized ferredoxin). The electron transfer pathway is presented in the direction of electron bifurcation although physiological function of Nfn is found to reduce NADP^+^.

Soon after its discovery, Nfn was studied as an ideal object to determine the mechanism of FBEB. A bacterial NfnAB (from *T. maritima*, PDB ID: 4YRY) and two archaeal NfnAB (from *P. furiosus*, PDB ID: 5JCA and 5VJ7), which represent the Pattern A mentioned above, are well characterized, including their crystal structures (Demmer et al., 2015; Lubner et al., 2017; Nguyen et al., 2017). Crystal structures reveal that NfnAB exists as a heterodimer, despite forming Nfn(AB)_2_ heterotetramer in solution state. The masses of these NfnAB are similar to those of *C. kluyveri* and they bind the same cofactors, including an FAD molecule and a [2Fe2S] cluster of NfnA, an FAD molecule and two [4Fe4S] clusters on NfnB (Figure 4).

**FIGURE 5:**
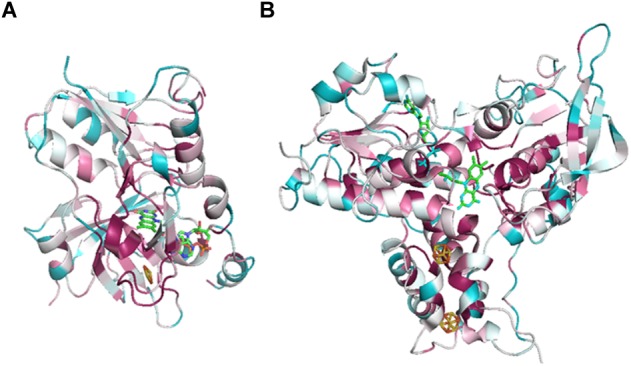
ConSurf analysis of NfnA **(A)** and NfnB **(B)** from *T. maritima* (PDB: 4YLF). Analysis was performed by ConSurf server (http://consurf.tau.ac.il/2016/), 150 aligned sequences of Nfn homologs are used for this analysis. Cofactors are depicted by sticks. Conserved sites are colored by pink and variant sites are colored by blue.

The amino acid sequence of Nfn is highly conserved. Approximately one-third of amino acid residues of Nfn are similar, particularly those near the cofactors (Figure 5). Further, the three crystal structures are nearly identical. NfnA, which belongs to the plant-type Fnr (ferredoxin:NADP^+^ reductase) protein family, consists of an antiparallel six-stranded β-barrel, an NAD-binding domain, and a C-terminal extension. The FAD-binding domain is highly similar to those of other Fnr members, in that FAD binds the β-barrel in a bent conformation. The isoalloxazine ring of FAD on NfnA (a-FAD) is hydrogen-bonded to a conserved strand (ERxPxT), where the threonine residue is hydrogen bonded to the N_5_ atom. Different from other Fnr members, in which the O-atom of isoalloxazine ring is generally bonded to a lysine residue, the O-atom of the FAD on NfnA is bonded to different residue in different species, such as arginine, glutamine, or leucine. The C-terminal extension of NfnA differs from that of Fnr, which is subdivided into a [2Fe2S] cluster binding domain and a long helical strand. The [2Fe2S] cluster binding domain is well conserved (DGTGMCGxCRx_10_CV), and the aspartic acid residue is atypical for iron–sulfur clusters, which leads to a higher redox potential (+80 mV) compared with the other four cysteine ligated [2Fe2S] clusters (Hagen et al., 2000).

NfnB consists of a helical domain containing two iron–sulfur clusters and two Rossmann folds that bind FAD and NADPH; and its structure is similar to that of NADPH-dependent glutamine synthase. The hydrophobic domain of the two [4Fe4S] clusters is well conserved with the motifs RCx_2_Cx_4_Cx_3_CPV and GRVCPQExQCx_3_Cx_13_Gx_2_ER. The sequences between the iron–sulfur clusters and the FAD-binding domain are more flexible, followed by a highly conserved FAD-binding domain. The isoalloxazine ring of FAD is located in the pocket of the three domains.

The NADH and NADPH binding sites of Nfn were determined from the crystal structures of *T. maritima* NfnAB and *P. furiosus* NfnI, respectively. NADH binds to a-FAD on NfnA, and NADPH binds to b-FAD on NfnB. The results of a docking test predict that ferredoxin binds near the distal [4Fe4S] cluster of NfnB (Demmer et al., 2015; Lubner et al., 2017). The NAD^+^ binding sites in NfnA are less conserved compared to those of other cofactors. In other members of Fnr family, to which the NfnA belongs, the phosphate group of NADP^+^ is hydrogen-bonded to several amino acid residues including arginine, lysine, serine, and threonine. In the NfnA homologs, these sites are replaced by the residues of aspartic acid, glycine, and two valines, respectively, which is not feasible for binding the NADP^+^. The NADP^+^ binding sites in NfnB are unambiguously identified by the structure of *P. furiosus* NfnI. In the NADP^+^ binding domain, two conserved arginine residues are hydrogen-bonded to the extra phosphate group of NADP^+^, and other residues for binding NADP^+^ are also conserved, which may contribute to the selectivity of the substrate NADP^+^.

There are features specific to different Nfn homologs. For example, the clostridial Nfn significantly differs from those of other species in the sequence between the a-FAD and NADH-binding domains. Specifically, the sequence of the clostridial Nfn is 7 amino acid residues longer than the others, and its amino acid composition is strikingly different. Another crucial difference is that the sequence of NADH-binding domain of NfnII of thermophilic archaea significantly differs from those of NfnI. A recent study suggested that the enzyme activity of NfnII of *P. furiosus* differs from that of NfnI, in that it shows no NAD^+^-dependent activity and might have a different high-potential electron acceptor (Nguyen et al., 2017). The difference in the primary sequence might change the structure of the NADH-binding domain of NfnII, which abrogates the binding of NAD^+^ to NfnA.

Research on the crystal structure of Nfn, together with the knowledge of its optical, paramagnetic spectroscopic, and electrochemical properties provide deep insights into the mechanism of electron bifurcation (Demmer et al., 2016; Berry et al., 2017; Lubner et al., 2017). It was revealed that three iron–sulfur clusters and two FAD molecules on NfnAB compose the electron transfer chain, where b-FAD is the site of electron bifurcation (Figure 4). The bifurcation reaction is started by the oxidation of NADPH at b-FAD, after which an electron is transferred to the [2Fe2S] cluster along the exergonic branch. The distance (14–15 Å) between the [2Fe2S] cluster and b-FAD is close to the longest distance for electron transfer (14 Å) (Page et al., 1999). Hydrogen-deuterium exchange mass spectrometry shows that an NfnA–NfnB rearrangement upon NADPH binding reduces the distance between the [2Fe2S] and b-FAD to approximately 13 Å and allows the electron transfer to the [2Fe2S] cluster and then to the NADH-binding site at a-FAD (Berry et al., 2017).

Along the endergonic branch of the reaction, the redox potentials of the two [4Fe4S] clusters are -513 mV and -718 mV, respectively (Lubner et al., 2017), indicating that electrons could transfer between the [4Fe4S] cluster and ferredoxin (-500 mV). Analysis using transient UV-Vis absorption spectroscopy (TAS) revealed a short-lived anionic semiquinone (ASQ), which could drive one electron to a reduced state sufficient to reduce the [4Fe4S] cluster (Lubner et al., 2017). Thus, electron transfer involved in the bifurcation reaction can be summarized as follows: firstly, NADPH (-370 mV) reduces the flavin quinone to hydroquinone (HQ, E_OX/HQ_ = -276 mV) with two electrons; then one electron leaves the hydroquinone (E_ASQ/HQ_ = +359 mV) and travels to [2Fe2S] (+80 mV) and further to a-FAD; the remaining anionic flavin semiquinone (E_OX/ASQ_ = -911 mV) immediately reduces the proximal [4Fe–4S] cluster (-718 mV) and the electron is further transferred to ferredoxin via the distal [4Fe4S] (Lubner et al., 2017; Buckel and Thauer, 2018a).

**FIGURE 6:**
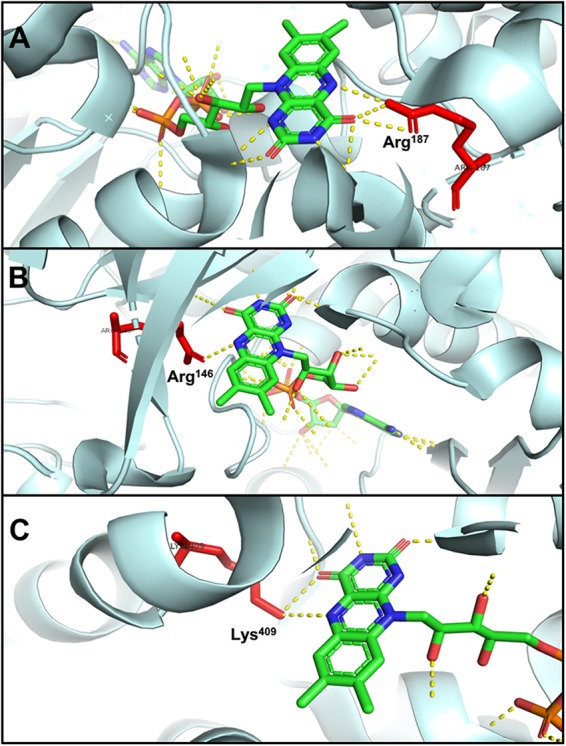
Hydrogen bonding in electron-bifurcating FADs. Arginine and lysine residues that form hydrogen bonds with FAD are red; in FAD, green, blue, orange, and yellow indicate carbon, nitrogen, oxygen, and phosphorus, respectively; the hydrogen bonds of FAD are shown as yellow dotted lines. **(A)** N_5_ of b-FAD binds an arginine residue (Arg^187^ of *T. maritima* NfnB, PDB: 4YRY). **(B)** N_5_ of FAD binds an arginine residue (Arg^146^ of *A. fermentans* EftA, PDB: 4KPU). **(C)** N_5_ of FAD binds a lysine residue (Lys^409^ of *Methanothermococcus thermolithotrophicus* HdrA, PDB: 5ODC).

The N_5_ atom of b-FAD, the site that receives electrons and protons transferred from NADPH, is hydrogen-bonded to an arginine residue (Figure 6A), which might obstruct protonation of the FAD semiquinone and inhibit the formation of low-energy neutral flavin semiquinone (NSQ) (Demmer et al., 2015; Lubner et al., 2017). The arginine residue bound to N_5_ of isoalloxazine is conserved in all Nfn homologs, indicating its importance to electron bifurcation. The arginine residue is predicted to play a key role in adjusting the low redox potential of the FADH^∗^/FAD pair required for ferredoxin reduction (Demmer et al., 2015). This conserved residue is also present in Bcd/EtfAB of *Acidaminococcus fermentans* (PDB ID: 4KPU), where the FAD of EtfB, the proposed electron bifurcation site, forms a hydrogen bond between an arginine residue of EtfA and the N_5_ of the flavin semiquinone (Figure 6B) (Chowdhury et al., 2014). This arginine residue is conserved in both bifurcating and non-bifurcating Etfs, and in the non-bifurcating Etfs, the site of isoalloxazine ring is empty (Buckel and Thauer, 2018a). In the archaeal bifurcating flavoprotein heterodisulfide reductase (HdrABC-MvhADG, PDB ID: 5ODC), the proposed bifurcating FAD of HdrA is hydrogen-bonded to a lysine (Figure 6C) (Wagner et al., 2017), that is also conserved among HdrA homologs. The structural similarity among different types of electron-bifurcating enzymes suggests that they share the same bifurcating mechanism on the flavin. In particular, the short-lived ASQ may be essential for all electron bifurcations. However, equilibrium and ultrafast kinetic studies of electron transfer suggest that a short-lived ASQ is important but not sufficient for electron bifurcation, because non-bifurcating flavoproteins also generate ASQ, and different mechanisms dominate ASQ decay in the different protein environments (Hoben et al., 2017).

## The Physiological Function of Nfn

### Nfn in Butyrate Formation of *C. kluyveri*

The physiological function of Nfn was first characterized in *C. kluyveri*, which uniquely ferments acetate and ethanol to butyrate, caproate, and H_2_. During this process, only NADH is produced from the oxidation of ethanol, while NADPH is required (3-hydroxy-butyryl CoA dehydrogenase) as well as NADH for the butyrate formation in the reductive part. Because genes are absent in the *C. kluyveri* genome that encode a proton-translocating transhydrogenase which catalyze the energy-dependent NADP^+^ reduction with NADH, there must be another transhydrogenase in *C. kluyveri*. Cell extracts of *C. kluyveri* were shown to catalyze the reduction of ferredoxin with NADPH only in the presence of NAD^+^, where NAD^+^ is simultaneously reduced (Jungermann et al., 1971). Not until 2010 were the two reactions shown to be catalyzed by the enzyme NfnAB through FBEB as one reaction (Wang et al., 2010). Physiologically, Nfn catalyzes the electron-confurcating NADP^+^ reduction with NADH and reduced ferredoxin since NADPH is needed in the metabolism. Therefore, the enzyme is also called ferredoxin-dependent transhydrogenase. Within the cells, ferredoxins are more than 90% reduced, with redox potentials ≤-500 mV. The NADH/NAD^+^ ratio is generally near 0.3, and the NADPH/NADP ratio is generally >1 (Decker and Pfitzer, 1972; Thauer et al., 1977; Bennett et al., 2009), and their redox potentials under physiological conditions are -280 mV and -370 mV, respectively. Therefore, the endergonic NADP^+^ reduction (ΔG’ = 17 kJ/mol) with NADH coupled to the exergonic NADP^+^ reduction with reduced ferredoxin (ΔG’ = -25 kJ/mol) can occur (totally ΔG’ = -8 kJ/mol). Energy is conserved via the reactions catalyzed by the electron-bifurcating Nfn and Bcd/EtfAB and the membrane-bound Rnf, and the reducing equivalents are balanced in *C. kluyveri*. Interestingly, in the genome of *C. kluyveri*, the *nfn* gene forms a large transcription unit *rex*-*crt1*-*bcd1*-*etfB1A1*-*hbd1-nfn* with the genes responsible for butyrate formation (Figure 2). Their expression is regulated by the global redox-sensing transcriptional regulator Rex according to the cellular level of NADH/NAD^+^, implicating that Nfn is directly involved in the butyrate fermentation (Hu et al., 2016).

### Nfn in Acetogenesis and CO_2_ Fixation in *M. thermoacetica* and *C. autoethanogenum*

Nfn performs functions in the acetogenesis of *M. thermoacetica* (Huang et al., 2012). When the homoacetogen grows heterotrophically on glucose, three acetic acid molecules can be produced. In the pathway, one glucose molecule is catabolized to two pyruvate molecules via classical glycolysis, which are further converted into two acetyl-CoA and two CO_2_. Finally, two acetic acid molecules are produced from acetyl-CoA, and one more is generated by two CO_2_ fixations via the Wood–Ljungdahl pathway (also called the acetyl-CoA pathway). During the metabolism of glucose, two NADH and two reduced ferredoxins are generated by glyceraldehyde-3-phosphate dehydrogenase and pyruvate:ferredoxin oxidoreductase, respectively. However, among six electrons required for CO_2_ reduction to the methyl carbon of acetate, four are provided by NADPH since both formate dehydrogenase and methylenetetrahydrofolate dehydrogenase are NADPH-dependent. The problem with the requirement for NADPH was solved by the identification of Nfn (Huang et al., 2012), which bridges CO_2_ fixation to glycolysis via its transhydrogenase activity and maintains redox balance *in vivo*. This mechanism is also supported by findings that *nfn* resides upstream of the gene encoding NADPH-dependent methylenetetrahydrofolate dehydrogenase (Figure 2).

*M. thermoacetica* also grows autotrophically on H_2_ and CO_2_. In this case, the reducing power required to reduce CO_2_ is acquired from the oxidation of hydrogen gas, and the electron-bifurcating [FeFe]-hydrogenase HydABC catalyzes the reduction of NAD^+^ and ferredoxin with hydrogen gas (Wang et al., 2013). Similar to the heterotrophic mechanism employed in glucose metabolism, the reducing equivalents reduce NADP^+^ through Nfn, thereby linking CO_2_ fixation to the oxidation of hydrogen gas. In the Wood–Ljungdahl pathway, one ATP molecule is consumed in the formation of 10-formyltetrahydrofolate from a formate, and one ATP is produced via the acetate formation from acetyl-phosphate via substrate-level phosphorylation. Thus, the net output of ATP is zero. The energy conservation in the reaction of Nfn, together with the electron-bifurcating HydABC and hexaheteromeric methylenetetrahydrofolate reductase and the membrane-bound Ech, helps the bacterium to survive in the energy-limited environment (Mock et al., 2014; Schuchmann and Müller, 2014).

The acetogen *C. autoethanogenum* harbors a fused Nfn. However, this putative enzyme remains to be characterized in detail (Wang et al., 2013; Mock et al., 2015). The prediction of the existence of this enzyme is based on the results of BLAST search and detection of Nfn activities in extracts of cells grown on fructose, CO, or H_2_ and CO_2_. Here, NADPH is also required for the Wood–Ljungdahl pathway. Nfn is predicted to catalyze either electron-bifurcating or electron-confurcating reactions, depending on growth conditions, where there are high activities of the electron-bifurcating NADP-dependent [FeFe]-hydrogenase/formate dehydrogenase complex FdhA/HytA-E. Thus, Nfn is predicted to contribute to energy conservation together with electron-bifurcating FdhA/HytA-E and the membrane-bound Rnf. The fused *nfn* gene resides downstream of the gene encoding the redox-sensing transcription regulator Rex, together with the genes for a predicted multimeric [FeFe]-hydrogenase (Figure 2).

### Deletion of *nfnAB* From *Thermoanaerobacterium saccharolyticum, D. alaskensis* G20, and *C. thermocellum*

The genes encoding NfnAB were first deleted from *T. saccharolyticum* (Lo et al., 2015). Deletion of *nfnAB* caused loss of NADPH-dependent ferredoxin oxidoreductase activity in cell extracts. In wild-type *T. saccharolyticum*, deletion of *nfnAB* causes a 46% increase in H_2_ formation but little change in other fermentation products. In two strains engineered to produce high yields of ethanol, loss of *nfnAB* caused the response as follows: one *ΔnfnAB* strain had a decreased ethanol yield and showed NADPH-dependent alcohol dehydrogenase (Adh) activity, while the other *ΔnfnAB* strain had unchanged ethanol yield and showed NADH-dependent Adh activity. These findings indicate that NfnAB plays a key role in generating NADPH in strains harboring NADPH-dependent Adh. Moreover, significant NADH-dependent ferredoxin oxidoreductase activity detected in cell extracts of all strains, which was derived from ferredoxin:NAD^+^ oxidoreductase (Fnor) (Tian et al., 2016), suggesting that Nfn functions in concert with Fnor and other oxidoreductases.

The deletion of *nfnAB* also slows the growth rate and decreases the magnitude of hydrogen isotopic fractionation of lipids and overall fractionation, which reflects the state of central energy metabolism (Zhang et al., 2009; Leavitt et al., 2017). The regulation of the ^2^H/^1^H ratio may be linked to the intracellular production of NAD(P)H. Therefore, the presence of an electron-bifurcating transhydrogenase might explain the complexity of the hydrogen isotopic fractionation in anaerobes. A similar overall trend in growth rate and fractionation, as well as the isotopic ordering of individual lipids, have been observed in the heterotrophic sulfate reducer, *D. alaskensis* G20, when deleting the *nfnAB* genes and growing on malate and fumarate (Leavitt et al., 2016). However, this phenotype is attenuated or absent when pyruvate is used, suggesting the functions of NfnAB vary according to nutritional conditions and available substrates. Further, deletion of *nfnAB* from *C. thermocellum* has little effect on the distribution of fermentation products, including ethanol production. These findings suggest that NfnAB may not play a major role in metabolism when cells are grown on cellobiose and Avicel. This is may be explained if it is not the NADPH-dependent enzymes play a key role in central metabolism and ethanol production but NADH-dependent ones such as Rnf, aldehyde dehydrogenase, and Adh in the bacterium (Lo et al., 2017).

### Two Functionally Different NfnAB of *P. furiosus*

A recent study on the archaea *P. furiosus* Nfn reveals different functions performed by two types of Nfn (Nguyen et al., 2017). The genome of *P. furiosus* harbors two copies of genes encoding NfnAB (NfnI and NfnII). Deletion or overexpression of *nfn*I and *nfn*II inhibits the growth of *P. furiosus* in the presence of different carbon and redox source (three culturing conditions: maltose only, maltose plus S^0^, and peptides plus S^0^), indicating that the moderate expression of Nfn is required for the robust growth of *P. furiosus*. In the absence of sulfur, the growth of *Δnfn*I and OE-*nfn*I (overexpression of *nfn*I) mutant is more severely inhibited, suggesting that NfnI may be required mainly for carbohydrate metabolism and is not essential in the presence of peptides as carbon source or sulfur as the terminal electron acceptor. Under the conditions of maltose plus S^0^ and peptides plus S^0^, the expression of *nfn*I decreases and that of *nfn*II increases, indicating that NfnII is required in the presence of sulfur or peptides. Moreover, the growth phenotype of *nfn*-deletion mutants of *P. furiosus* may be caused by the redox unbalance, because deletion of either of the *nfn* genes causes the increase of total amount of NAD(H) and NADP(H) and the decrease of the ratio of NADPH/NADP. The change is more apparent in the *Δnfn*I strain in the absence of sulfur, suggesting that in the absence of sulfur, NfnI is the key enzyme required for producing NADPH from NADH and reduced ferredoxin. Consistent with this observation, the expression of L-aspartate oxidase, the first enzyme of the NAD^+^ salvage pathway, are significantly decreased in the *Δnfn*I strain, which could avoid the NADH accumulation. NfnII may have a more important role in the presence of sulfur, but its function is still not clear. NfnII has shown a NAD^+^-independent ferredoxin:NADP^+^ oxidoreductase activity, whether it is a non-bifurcating enzyme or it has another substrate to replace NAD^+^ is unknown (Nguyen et al., 2017).

## Perspectives

FBEB is a novel mechanism involved in energy metabolism in anaerobes. The electron-bifurcating transhydrogenase Nfn has appealed increasing focus as a model to understand the function and mechanism of FBEB. The wide distribution and intriguing physiological functions of Nfn indicate that this enzyme plays important roles in anaerobic metabolism. To completely understand this special enzyme and FBEB, further studies on several aspects are still needed.

(1) More evidences are still needed to confirm the origin and evolution of Nfn. Nfn distributed widely in both bacteria and archaea, especially in the arguably ancient anaerobes, methanogens and acetogens. Evidence indicates that acetogenesis and methanogenesis were ancient metabolic pathways in the nutritionally deficient environment (Martin et al., 2014). It has been shown that Nfn, together with other electron-bifurcating enzymes, play a vital role in the metabolism of acetogenesis (Schuchmann and Müller, 2014). Thus, it is reasonable to suppose that Nfn is a property of the LUCA. However, a recent study showed that Nfn originated from a group of recent evolved Actinobacteria and Proteobacteria and spread by lateral gene transfer (Poudel et al., 2018), which is surprising when considering that Nfn is widely distributed in the early evolved bacteria and archaea. So, further investigations are in need to reveal the evolution of this electron-bifurcating transhydrogenase.

(2) The biochemical properties and physiological functions of different Nfn homologs need further research. Most studies of Nfn have focused on the homologs that belong to the Pattern A, while detailed properties of homologs of other three patterns are still not clear. Evidences indicate that NfnI and NfnII of *P. furiosus*, which both belong to the Pattern A and show little differences in sequence (70% identity) and structure, perform different functions under different environments (Nguyen et al., 2017). Thus, the Nfn homologs may catalyze different reactions (bifurcating or non-bifurcating) and have diverse physiological functions, and we could not tell their functions only from their sequences. Some specific Nfn homologs may need more attention, such as the homologs of the aerobic *Candidatus Koribacter versatilis* (Pattern A), Actinobacteria species like *Mobiluncus curtisii* (Pattern C), and the eukaryotic *S. salmonicida* (Pattern D). It is also noteworthy that functions of archaeal NfnIII (Pattern A), and the Nfn homologs from methanogens (Pattern A) and Bacteroidetes (Pattern B) have not been studied. Moreover, published studies were performed in laboratory using pure cultures. Therefore, further studies are required to determine how the enzyme functions in natural microbial communities and in other special niches such as in the gut microbiota and in the anaerobic fermentation of waste. Meta-omics studies and analysis of enzyme activity *in situ* will likely reveal the role of Nfn in the ecological interactions of anaerobes with their environments.

(3) Further structural and mechanistic studies on Nfn may enhance our understanding of FBEB. Besides Nfn, there are also other flavoenzymes that possess electron bifurcation activity. These can be classified into structurally different groups as follows (Buckel and Thauer, 2018b): (1) EtfAB-containing complexes, (2) NAD(P)H dehydrogenase-containing complexes, (3) heterodisulfide reductase (HdrABC)-containing complexes. In contrast, Nfn is unique in that its structure is simpler and unrelated to those of the three groups (Buckel and Thauer, 2018a). With the help of physicochemical technologies such as TAS, we now learn that the short-lived ASQ is necessary for electron bifurcation. However, recent research performed with TAS suggests that the short-lived ASQ may be insufficient to explain FBEB (Hoben et al., 2017). Thus, thermodynamic and kinetic studies are still required to understand the detailed mechanism of electron transfer. Considering the structural similarity in flavin binding, the mechanism of electron transfer employed by Nfn may provide new clues that will help to understand other types of electron-bifurcating reactions.

(4) Nfn might be a potential approach for redox engineering. In the metabolic engineering of industrial microorganisms, modification and introduction of metabolic pathways often cause fluctuations in the redox state, which may inhibit the cell growth and biosynthesis (Liu et al., 2018). Expression of pyridine nucleotide transhydrogenase (Pnt), which is a ubiquitous transhydrogenase in many microbes, is one of the most promising strategies (Liu et al., 2018) in redox engineering. Nfn provides an alternative engineering approach to regulate the redox status of anaerobes, where ferredoxin is another critical redox cofactor as well as NADH and NADPH. Considering that the reaction catalyzed by Nfn is reversible and energy-independent, engineering approach employing Nfn may offer more advantages in certain circumstance.

## Author Contributions

SW contributed the conception and design of the study. JL wrote the first draft of the manuscript. JL, HH, and SW wrote sections of the manuscript. All authors contributed to manuscript revision, read and approved the submitted version.

## Conflict of Interest Statement

The authors declare that the research was conducted in the absence of any commercial or financial relationships that could be construed as a potential conflict of interest.

## References

[B1] Andersson J. O. Roger A. J. (2002). Evolutionary analyses of the small subunit of glutamate synthase: gene order conservation, gene fusions, and prokaryote-to-eukaryote lateral gene transfers. *Eukaryt. Cell* 1 304–310. 10.1128/EC.1.2.304-310.2002 12455964 PMC118040

[B2] Bennett B. D. Kimball E. H. Gao M. Osterhout R. Van Dien S. J. Rabinowitz J. D. (2009). Absolute metabolite concentrations and implied enzyme active site occupancy in *Escherichia coli*. *Nat. Chem. Biol.* 5 593–599. 10.1038/nchembio.186 19561621 PMC2754216

[B3] Berry L. Poudel S. Tokmina-Lukaszewska M. Colman D. R. Nguyen D. M. N. Schut G. J. (2017). H/D exchange mass spectrometry and statistical coupling analysis reveal a role for allostery in a ferredoxin-dependent bifurcating transhydrogenase catalytic cycle. *Biochim. Biophys. Acta Gen. Subj.* 1862 9–17. 10.1016/j.bbagen.2017.10.002 28993252

[B4] Buckel W. Thauer R. K. (2013). Energy conservation via electron bifurcating ferredoxin reduction and proton/Na^+^ translocating ferredoxin oxidation. *Biochim. Biophys. Acta Bioenerg.* 1827 94–113. 10.1016/j.bbabio.2012.07.002 22800682

[B5] Buckel W. Thauer R. K. (2018a). Flavin-based electron bifurcation, a new mechanism of biological energy coupling. *Chem. Rev.* 118 3862–3886. 10.1021/acs.chemrev.7b00707 29561602

[B6] Buckel W. Thauer R. K. (2018b). Flavin-based electron bifurcation, ferredoxin, flavodoxin, and anaerobic respiration with protons (Ech) or NAD^+^ (Rnf) as electron acceptors: a historical review. *Front. Microbiol.* 9:401. 10.3389/fmicb.2018.00401 29593673 PMC5861303

[B7] Chowdhury N. P. Mowafy A. M. Demmer J. K. Upadhyay V. Koelzer S. Jayamani E. (2014). Studies on the mechanism of electron bifurcation catalyzed by electron transferring flavoprotein (Etf) and butyryl-CoA dehydrogenase (Bcd) of *Acidaminococcus* fermentans. *J. Biol. Chem.* 289 5145–5157. 10.1074/jbc.M113.521013 24379410 PMC3931072

[B8] Decker K. Pfitzer S. (1972). Determination of steady-state concentrations of adenine nucleotides in growing *C. kluyveri* cells by biosynthetic labeling. *Anal. Biochem.* 50 529–539. 10.1016/0003-2697(72)90063-2 4345788

[B9] Demmer J. K. Huang H. Wang S. Demmer U. Thauer R. K. Ermler U. (2015). Insights into flavin-based electron bifurcation via the NADH-dependent reduced ferredoxin:NADP oxidoreductase structure. *J. Biol. Chem.* 290 21985–21995. 10.1074/jbc.M115.656520 26139605 PMC4571952

[B10] Demmer J. K. Pal Chowdhury N. Selmer T. Ermler U. Buckel W. (2017). The semiquinone swing in the bifurcating electron transferring flavoprotein/butyryl-CoA dehydrogenase complex from Clostridium difficile. *Nat. Commun.* 8:1577. 10.1038/s41467-017-01746-3 29146947 PMC5691135

[B11] Demmer J. K. Rupprecht F. A. Eisinger M. L. Ermler U. Langer J. D. (2016). Ligand binding and conformational dynamics in a flavin-based electron-bifurcating enzyme complex revealed by hydrogen–deuterium exchange mass spectrometry. *FEBS Lett.* 590 4472–4479. 10.1002/1873-3468.12489 27889905

[B12] Hagen W. R. Silva P. J. Amorim M. A. Hagedoorn P. L. Wassink H. Haaker H. (2000). Novel structure and redox chemistry of the prosthetic groups of the iron-sulfur flavoprotein sulfide dehydrogenase from *Pyrococcus furiosus*; evidence for a [2Fe-2S] cluster with Asp(Cys)3 ligands. *J. Biol. Inorg. Chem.* 5 527–534. 10.1007/PL00021452 10968624

[B13] Herrmann G. Jayamani E. Mai G. Buckel W. (2008). Energy conservation via electron-transferring flavoprotein in anaerobic bacteria. *J. Bacteriol.* 190 784–791. 10.1128/JB.01422-07 18039764 PMC2223574

[B14] Hoben J. P. Lubner C. E. Ratzloff M. W. Schut G. J. Nguyen D. M. N. Hempel K. W. (2017). Equilibrium & ultrafast kinetic studies manipulating electron transfer: a short-lived flavin semiquinone is not sufficient for electron bifurcation. *J. Biol. Chem.* 292 14039–14049. 10.1074/jbc.M117.794214 28615449 PMC5572931

[B15] Hu L. Huang H. Yuan H. Tao F. Xie H. Wang S. (2016). Rex in *Clostridium kluyveri* is a global redox-sensing transcriptional regulator. *J. Biotechnol.* 233 17–25. 10.1016/j.jbiotec.2016.06.024 27373958

[B16] Huang H. Wang S. Moll J. Thauer R. K. (2012). Electron bifurcation involved in the energy metabolism of the acetogenic bacterium *Moorella thermoacetica* growing on glucose or H2 plus CO2. *J. Bacteriol.* 194 3689–3699. 10.1128/JB.00385-12 22582275 PMC3393501

[B17] Jia Y. Ng S. K. Lu H. Cai M. Lee P. K. H. (2018). Genome-centric metatranscriptomes and ecological roles of the active microbial populations during cellulosic biomass anaerobic digestion. *Biotechnol. Biofuels* 11:117. 10.1186/s13068-018-1121-0 29713376 PMC5911951

[B18] Jungermann K. Rupprecht E. Ohrloff C. Thauer R. K. Decker K. (1971). Regulation of the reduced nicotinamide regulation of the reduced nicotinamide adenine reductase system in *Clostridium kluyveri*. *J. Biol. Chem.* 246 960–963. 5543695

[B19] Lawson P. A. Citron D. M. Tyrrell K. L. Finegold S. M. (2016). Reclassification of *Clostridium difficile* as *Clostridioides difficile* (Hall and O’Toole 1935). *Anaerobe* 40 95–99. 10.1016/j.anaerobe.2016.06.008 27370902

[B20] Leavitt W. D. Flynn T. M. Suess M. K. Bradley A. S. (2016). Transhydrogenase and growth substrate influence lipid hydrogen isotope ratios in *Desulfovibrio alaskensis* G20. *Front. Microbiol.* 7:918. 10.3389/fmicb.2016.00918 27445998 PMC4916218

[B21] Leavitt W. D. Murphy S. J. Lynd L. R. Bradley A. S. (2017). Hydrogen isotope composition of *Thermoanaerobacterium saccharolyticum* lipids: comparing wild type with a nfn-transhydrogenase mutant. *Org. Geochem.* 113 239–241. 10.1016/j.orggeochem.2017.06.020

[B22] Ley R. E. Turnbaugh P. J. Klein S. Gordon J. I. (2006). Human gut microbes associated with obesity. *Nature* 444:1022. 10.1038/4441022a 17183309

[B23] Li F. Hinderberger J. Seedorf H. Zhang J. Buckel W. Thauer R. K. (2008). Coupled ferredoxin and crotonyl coenzyme A (CoA) reduction with NADH catalyzed by the butyryl-CoA dehydrogenase/Etf complex from *Clostridium kluyveri*. *J. Bacteriol.* 190 843–850. 10.1128/JB.01417-07 17993531 PMC2223550

[B24] Liu J. Li H. Zhao G. Caiyin Q. Qiao J. (2018). Redox cofactor engineering in industrial microorganisms: strategies, recent applications and future directions. *J. Ind. Microbiol. Biotechnol.* 45 313–327. 10.1007/s10295-018-2031-7 29582241

[B25] Lo J. Olson D. G. Murphy S. J. L. Tian L. Hon S. Lanahan A. (2017). Engineering electron metabolism to increase ethanol production in *Clostridium thermocellum*. *Metab. Eng.* 39 71–79. 10.1016/j.ymben.2016.10.018 27989806

[B26] Lo J. Zheng T. Olson D. G. Ruppertsberger N. Tripathi S. A. Tian L. (2015). Deletion of nfnAB in *Thermoanaerobacterium saccharolyticum* and its effect on metabolism. *J. Bacteriol.* 197 2920–2929. 10.1128/JB.00347-15 26124241 PMC4542167

[B27] Lubner C. E. Jennings D. P. Mulder D. W. Schut G. J. Zadvornyy O. A. Hoben J. P. (2017). Mechanistic insights into energy conservation by flavin-based electron bifurcation. *Nat. Chem. Biol.* 13 655–659. 10.1038/nchembio.2348 28394885 PMC7646311

[B28] Ma K. Adams M. W. W. (1994). Sulfide dehydrogenase from the hyperthermophilic Archaeon *Pyroccoccus furiosus*: a new enzyme involved in the reduction of elemental sulfur. *J. Bacteriol.* 176 6509–6517. 10.1128/jb.176.21.6509-6517.1994 7961401 PMC197004

[B29] Ma K. Adams M. W. W. (2001). Ferredoxin: NADP oxidoreducatse from *Pyrococcus furiosus*. *Methods Enzymol.* 334 40–45. 10.1016/S0076-6879(01)34456-7 11398480

[B30] Martin W. Sousa F. Lane N. (2014). Energy at life’s origin. *Science* 344 1092–1093. 10.1111/soru.12033.11 24904143

[B31] Mccarver A. C. Lessner D. J. (2014). Molecular characterization of the thioredoxin system from *Methanosarcina acetivorans*. *FEBS J.* 281 4598–4611. 10.1111/febs.12964 25112424 PMC4199913

[B32] Metcalf W. W. (2016). Classic spotlight: electron bifurcation, a unifying concept for energy conservation in anaerobes. *J. Bacteriol.* 198 1358–1358. 10.1128/JB.00185-16 27080054 PMC4836225

[B33] Mock J. Wang S. Huang H. Kahnt J. Thauer R. K. (2014). Evidence for a hexaheteromeric methylenetetrahydrofolate reductase in *Moorella thermoacetica*. *J. Bacteriol.* 196 3303–3314. 10.1128/JB.01839-14 25002540 PMC4135698

[B34] Mock J. Zheng Y. Mueller A. P. Ly S. Tran L. Segovia S. (2015). Energy conservation associated with ethanol formation from H2 and CO2 in *Clostridium autoethanogenum* involving electron bifurcation. *J. Bacteriol.* 197 2965–2980. 10.1128/JB.00399-15 26148714 PMC4542177

[B35] Nelson K. E. Clayton R. A. Gill S. R. Gwinn M. L. Dodson R. J. Haft D. H. (1999). Evidence for lateral gene transfer between Archaea and bacteria from genome sequence of *Thermotoga maritima*. *Nature* 399 323–329. 10.1038/20601 10360571

[B36] Nguyen D. M. N. Schut G. J. Zadvornyy O. A. Tokmina-Lukaszewska M. Poudel S. Lipscomb G. L. (2017). Two functionally distinct NADP^+^-dependent ferredoxin oxidoreductases maintain the primary redox balance of *Pyrococcus furiosus*. *J. Biol. Chem.* 292 14603–14616. 10.1074/jbc.M117.794172 28705933 PMC5582851

[B37] Nitschke W. Russell M. J. (2011). Redox bifurcations: mechanisms and importance to life now, and at its origin. *Bioessays* 34 106–109. 10.1002/bies.201100134 22045626

[B38] Page C. C. Moser C. C. Chen X. Dutton P. L. (1999). Natural engineering principles of electron tunnelling in biological oxidation-reduction. *Nature* 402 47–52. 10.1038/46972 10573417

[B39] Pereira I. A. C. Ramos A. R. Grein F. Marques M. C. da Silva S. M. Venceslau S. S. (2011). A comparative genomic analysis of energy metabolism in sulfate reducing bacteria and archaea. *Front. Microbiol.* 2:69 10.3389/fmicb.2011.00069 PMC3119410 21747791

[B40] Peters J. W. Miller A. F. Jones A. K. King P. W. Adams M. W. W. (2016). Electron bifurcation. *Curr. Opin. Chem. Biol.* 31 146–152. 10.1016/j.cbpa.2016.03.007 27016613

[B41] Poudel S. Dunham E. C. Lindsay M. R. Amenabar M. J. Fones E. M. Colman D. R. (2018). Origin and evolution of flavin-based electron bifurcating enzymes. *Front. Microbiol.* 9:1762. 10.3389/fmicb.2018.01762 30123204 PMC6085437

[B42] Sait M. Hugenholtz P. Janssen P. H. (2002). Cultivation of globally distributed soil bacteria from phylogenetic lineages previously only detected in cultivation-independent surveys. *Environ. Microbiol.* 4 654–666. 10.1046/j.1462-2920.2002.00352.x 12460273

[B43] Schuchmann K. Müller V. (2014). Autotrophy at the thermodynamic limit of life? a model for energy conservation in acetogenic bacteria. *Nat. Rev. Microbiol.* 12 809–821. 10.1038/nrmicro3365 25383604

[B44] Thauer R. K. Jungermann K. Decker K. (1977). Energy conservation in chemotrophic anaerobic bacteria. *Bacteriol. Rev.* 41 100–180. 860983 10.1128/br.41.1.100-180.1977 PMC413997

[B45] Tian L. Lo J. Shao X. Zheng T. Olson D. G. Lynd L. R. (2016). Ferredoxin: NAD^+^ oxidoreductase of *Thermoanaerobacterium saccharolyticum* and its role in ethanol formation. *Appl. Environ. Microbiol.* 82 7134–7141. 10.1128/AEM.02130-16 27694237 PMC5118924

[B46] Wagner T. Koch J. Ermler U. Shima S. (2017). Methanogenic heterodisulfide reductase (HdrABC-MvhAGD) uses two noncubane [4Fe-4S] clusters for reduction. *Science* 357 699–703. 10.1126/science.aan0425 28818947

[B47] Wang S. Huang H. Kahnt H. H. Mueller A. P. Köpke M. Thauer R. K. (2013). NADP-Specific electron-bifurcating [FeFe]-hydrogenase in a functional complex with formate dehydrogenase in *Clostridium autoethanogenum* grown on CO. *J. Bacteriol.* 195 4373–4386. 10.1128/JB.00678-13 23893107 PMC3807470

[B48] Wang S. Huang H. Moll J. Thauer R. K. (2010). NADP^+^ reduction with reduced ferredoxin and NADP^+^ reduction with NADH are coupled via an electron-bifurcating enzyme complex in *Clostridium kluyveri*. *J. Bacteriol.* 192 5115–5123. 10.1128/JB.00612-10 20675474 PMC2944534

[B49] Xu F. Jerlström-Hultqvist J. Einarsson E. Astvaldsson A. Svärd S. G. Andersson J. O. (2014). The genome of spironucleus salmonicida highlights a fish pathogen adapted to fluctuating environments. *PLoS Genet.* 10:e1004053. 10.1371/journal.pgen.1004053 24516394 PMC3916229

[B50] Zhang X. Gillespie A. L. Sessions A. L. (2009). Large D/H variations in bacterial lipids reflect central metabolic pathways. *Proc. Natl. Acad. Sci. U.S.A.* 106 12580–12586. 10.1073/pnas.0903030106 19617564 PMC2722351

